# A Comprehensive Review of Contemporary Role of Local Treatment of the Primary Tumor and/or the Metastases in Metastatic Prostate Cancer

**DOI:** 10.1155/2014/501213

**Published:** 2014-11-17

**Authors:** Fouad Aoun, Alexandre Peltier, Roland van Velthoven

**Affiliations:** ^1^Department of Urology, Jules Bordet Institute, 1 Héger-Bordet Street, 1000 Brussels, Belgium; ^2^Université Libre de Bruxelles, 50 Franklin Roosevelt Avenue, 1050 Brussels, Belgium

## Abstract

To provide an overview of the currently available literature regarding local control of primary tumor and oligometastases in metastatic prostate cancer and salvage lymph node dissection of clinical lymph node relapse after curative treatment of prostate cancer. *Evidence Acquisition*. A systematic literature search was conducted in 2014 to identify abstracts, original articles, review articles, research articles, and editorials relevant to the local control in metastatic prostate cancer. *Evidence Synthesis*. Local control of primary tumor in metastatic prostate cancer remains experimental with low level of evidence. The concept is supported by a growing body of genetic and molecular research as well as analogy with other cancers. There is only one retrospective observational population based study showing prolonged survival. To eradicate oligometastases, several options exist with excellent local control rates. Stereotactic body radiotherapy is safe, well tolerated, and efficacious treatment for lymph node and bone lesions. Both biochemical and clinical progression are slowed down with a median time to initiate ADT of 2 years. Salvage lymph node dissection is feasible in patients with clinical lymph node relapse after local curable treatment. *Conclusion*. Despite encouraging oncologic midterm results, a complete cure remains elusive in metastatic prostate cancer patients. Further advances in imaging are crucial in order to rapidly evolve beyond the proof of concept.

## 1. Introduction

In USA, prostate cancer is the most frequently diagnosed non-skin cancer in men and is second only to lung cancer as a cause of cancer deaths among men [1]. In 2014, it is estimated that 233 000 men in the United States will be diagnosed with a prostate cancer and 29 480 men will die from their disease [1]. Historically, approximately 25% of men presented with either regional or distant metastatic prostate cancer [2]. However, in the PSA era, a dramatic stage migration had resulted in proportionally more men being diagnosed at early stages, while the tumour is still organ confined, for which a treatment with curative intention is possible [2]. Nevertheless, <5% of patients will be diagnosed with synchronous metastatic disease and up to 40% will develop biochemical recurrence after conventional radical therapy [2, 3].

Traditionally, immediate or deferred androgen deprivation therapy (ADT) is offered as a palliative treatment to these patients to delay progression, curtail disease-related symptoms, and prolong survival. However, resistance to castration ultimately develops and despite the introduction of novel systemic agents, five-year survival for men with metastatic prostate cancer is only 28% [4]. Furthermore, these treatments are associated with significant morbidity and even mortality.

Recent advances in molecular and clinical imaging techniques have allowed the identification of an intermediate state where the cancer has spread outside the prostate gland but is not considered extensive in number and organ sites. This distinct state, termed oligometastatic state, was first described in a paper written in 1995 by Hellman and Weichselbaum [5]. However, even if this paper was not written specifically about prostate cancer, its relevance to prostate cancer is undeniable. Fortunately, their original concepts were revisited in 2011, suggesting that the evolution of metastatic capacity has intermediate states in which spread may be limited to specific organs and metastases might be present in limited numbers [6]. The clinical implication of this hypothesis is that localized forms of cancer treatment may be effective in patients with oligometastases.

For example, local control of the primary tumor itself has been associated with improved survival in patients diagnosed with metastatic glioblastoma [7], colon cancer [8], and renal cell carcinoma [9, 10]. In addition, decreasing tumor burden, through maximal cytoreductive surgery, radiation, or both, improves survival in patients with breast cancer [11], colon cancer [12], and ovarian cancer [13], in addition to increasing tumor response to systemic chemotherapy. Emerging data from prostate cancer indicate that maximum local treatment of the primary tumor prolongs progression-free survival (PFS), cancer-specific survival (CSS), and overall survival (OS). Furthermore, local treatment of the metastasis by surgical removal or by high-dose stereotactic radiotherapy can be beneficial to delay progression and to delay the initiation of systemic treatments. This novel idea is gaining interest in many prostate cancer centres, while others remain reluctant which further points out the necessity of working out an optimal treatment strategy to rapidly evolve beyond the proof of concept.

We performed a comprehensive review of the literature in order to analyze the evidence of the role and clinical outcome of local treatment of the primary tumor and/or the metastases in metastatic prostate cancer.

## 2. Evidence Acquisition

We performed a PubMed, Embase, and MEDLINE literature search in June 2014 using the keywords: prostate cancer, salvage lymphadenectomy, salvage lymph node dissection, salvage lymph node excision, oligometastases, low volume metastases, and stereotactic radiotherapy. To identify publications that address local control of the primary tumor in metastatic prostate cancer, the following keywords were used: metastatic prostate cancer AND prostatectomy and metastatic prostate cancer AND radiotherapy. For publications on recent developments in imaging techniques and their role in the management of patients with biochemical recurrence, additional sources were gathered by including the following keywords: positron emission tomography, choline, PSMA, computed tomography, magnetic resonance imaging, diffusion weighted MRI, and all these terms in combination with prostate cancer and biochemical recurrence. All titles were screened, and studies were excluded if obviously irrelevant. Abstracts were then examined and if necessary the full text was examined. Significant results and citations were reviewed manually by the authors. Articles published between 2000 and 2014 were reviewed and selected with the consensus of all the authors. We searched also the abstracts of ASCO and EAU conferences in urology in 2014.

## 3. Evidence Synthesis

### 3.1. Local Treatment of the Primary Tumor in Metastatic Prostate Cancer: The Paradigm Shift

Historically, radical prostatectomy was avoided for patients with locally advanced prostate cancer presumed to have extraprostatic disease and these patients were offered radiation therapy. However, external irradiation alone in locally advanced prostate cancer was associated with poor long-term oncologic outcome. Oncological failure was due to the presence of undetectable micrometastases outside the planning target volume. In an attempt to improve cancer control, a combination of androgen suppression and external irradiation was used in the mid-1980 to destroy hormone-dependent micrometastases. The benefit of the addition of long-term adjuvant ADT to local radiotherapy in patients with locally advanced prostate cancer was first demonstrated in 1997 [14, 15]. Results from the European Organisation for Research and Treatment of Cancer (EORTC) 22863 and the Radiation Therapy Oncology Group (RTOG) 85-31 trials demonstrated significant improvements in local and distant disease control with combination therapy; a benefit in 10-year CSS and OS was later confirmed [16, 17]. Two recent meta-analyses confirmed these results [18, 19]. Since then, combination of androgen suppression with external irradiation had become the standard of treatment of locally advanced prostate cancer with a high level of evidence (level 1, grade A) [20]. Androgen suppression provides a method to improve the outcome of external irradiation alone, possibly by eliminating occult systemic disease. Moreover, androgen suppression and external irradiation seem to have an additive effect on local control by induction of apoptosis [21]. Adjuvant ADT is also effective with surgery in node positive disease. In one prospective randomised study, PFS, CSS, and OS were improved in the group treated with long-term adjuvant hormonal treatment after radical prostatectomy with pelvic lymph node dissection (PLND) compared to after surgery alone [22].

In the last decade, a new emerging concept, based on the primary control of the tumor rather than systemic treatment by ADT in micrometastatic disease, had gained place due to three randomized control trials. Widmark et al. reported a cancer-specific and overall mortality decrease by 12% and 9.8%, respectively, with combined ADT and radiotherapy compared with ADT alone at 10 years [23]. The National Cancer Institute of Canada-Clinical Trials Group/Southwest Oncology Group T94-0110 trial also showed a significant reduction in the cancer-specific and overall mortality risk with the addition of radiotherapy to ADT after a median follow-up of 6 years [24]. While a slight increase in overall bother from urinary and bowel symptoms may occur from combined therapy, the associated risk-to-benefit ratio remained favourable for combined therapy in these studies. In another randomized trial, the addition of radiation to hormone therapy had led also to a significant improvement in 5-year locoregional control and metastases-free progression [25]. In view of these randomised controlled trials, local control of primary tumor by radiotherapy associated with ADT significantly reduces the risk of progression and improves locoregional control in patients with locally advanced prostate cancer and is now considered a standard treatment option.

In a systematic review of randomised studies, published in 2010, convincing evidence of a survival benefit following local treatment in high-risk localised or locally advanced disease was reported, including patients with node-positive disease after radical prostatectomy [26].

In a large retrospective analysis, increasing radiation doses resulted in better locoregional control and improved metastasis-free survival [27]. Improved survival is likely due to a reduced incidence of late distant metastases developing as a result of locoregional progression.

A recent retrospective analysis demonstrating a significant survival advantage for both overall and relative survival in node positive patients who had undergone radical prostatectomy is in line with this hypothesis highlighting the importance of locoregional control in micrometastatic patients [28]. In contrast, few data exist regarding the impact on survival of local control of the primary tumor in metastatic prostate cancer. There is no prospective data regarding a survival benefit for patients with metastatic prostate cancer undergoing treatment of the primary tumor. However, there are retrospective studies that do indicate improved outcome with prostate tumor cytoreduction [25–30]. In a match pair analysis of 79 patients with node positive prostate cancer, Ghavamian et al. found that patients undergoing radical prostatectomy with PLND and orchiectomy demonstrated higher disease specific and overall survivals compared to patients undergoing PLND and orchiectomy alone [29]. Furthermore, in studies evaluating systemic treatment of metastatic prostate cancer, an increased response was found in patients who had undergone prior radical prostatectomy [31–33]. Qin et al. examined cytoreductive surgery in patients with newly diagnosed metastatic prostate cancer treated with complete androgen blockade who underwent transurethral resection of the prostate in comparison with patients without TURP. At a median follow-up of 15 months, prostate-specific antigen (PSA) kinetics and PFS significantly favored the TURP group. CSS and OS tended toward significance in favor of the TURP group [34]. Finally, there are two population based studies, the first using the Munich cancer registry and the second the SEER database, suggesting a possible survival benefit of primary treatment of the prostate in men diagnosed with metastatic prostate cancer. In the first, Engel et al. demonstrated that patients undergoing radical prostatectomy in node positive disease had a higher 10-year OS and CSS compared with patients undergoing only PLND [28]. In the second, Culp et al. examined 8185 patients with metastatic prostate cancer. They demonstrated that patients undergoing definitive treatment of the prostate had a higher 5-year OS and DSS probability compared with patients not undergoing local therapy [35]. In addition, local control of primary tumor is the only modality delivered with intention to eradicate local disease because studies have shown that systemic treatment had an unacceptable failure rate to control the tumor locally. Postradiotherapy prostate biopsies performed following primary ADT reveal a high rate of persistence of local disease [36]. In the SPCG-7 trial, the postradiation therapy biopsy positivity rate was 66% [36]. Finally, it is noteworthy to mention that the benefit of local control is immediate by treating problems resulting from uncontrolled locally advanced disease such as lower or upper urinary tract obstruction, macroscopic hematuria, LUTS, and rectal irritation [37].

To date, local control of the primary tumor in metastatic prostate cancer remains experimental with a low level of evidence because of small patient numbers, limited follow-up, and the retrospective design of the studies. Well-designed, multicenter, prospective, and randomized controlled studies are required to definitely establish the role of primary control as standard of care in metastatic prostate cancer and to select patients eligible to such a therapy.

### 3.2. Local Treatment of the Primary Tumor in Metastatic Prostate Cancer: The Abscopal Effect versus the Fisher and Folkman Effects

The primary prostate tumor, its host, and regional and distant metastases are communicating ecosystems, characterised by a complex connecting network of host cells and molecular pathways. As a consequence, manipulation of the primary tumour may have beneficial, detrimental, or no effect on the other elements. In prostate cancer, little is known on how local control of the primary tumor affects the established communicating ecosystem. Emerging data and analogy with other types of cancer indicate possible benefit of treating the primary tumor in metastatic prostate cancer patient (abscopal effect). However, the mechanisms underlying the survival benefit of cytoreductive treatment in metastatic prostate cancer remain enigmatic. For some authors, removing tumor-promoting factors and immunosuppressive cytokines and decreasing the total tumor burden allow for an improved response to ADT and/or chemotherapy by eliminating the primary source of the dissemination of metastatic cells through the seed and soil unidirectional hypothesis [38]. Recently, the proven ability of cancer cells to seed not only to regional and distant sites in the body but also to the tumor itself is a new concept rapidly evolving in cancer research [39]. By eliminating the primary site, a better local control is achieved through the self-seeding multidirectional hypothesis [39]. For others, intratumoral synthesis of testosterone from weak adrenal androgens is a substantial source of intraprostatic androgen following ADT [40]. This synthesis may protect primary prostate cancer cells from ADT and provide a sanctuary for prostate cancer cells to progress to castrate resistance [40]. These castrate resistant clones may also be present in the prostate prior to the initiation of ADT and they could be enriched through clonal selection after testosterone decline [41]. This theory is supported by animal models demonstrating that the use of early local treatment eliminates androgen independent clones with the potential to delay time to castrate resistance and hence prolong disease control [42, 43]. In the same way, it was also hypothesized that local failures can lead to a second wave of distant metastases [44]. In addition, the parallel with breast cancer stems from the idea that both cancers are hormone sensitive with a long-term outcome improved by combination of radiotherapy and hormonal treatment, and long-term adjuvant hormonal treatment significantly improves OS as well as treatment of the primary prostate and breast tumor in patients who present with metastatic disease. In contrast to breast cancers where multiple of clinical studies explore existing paradigms on the capacity of metastatic spread and assess the role of local control of the primary tumor, few studies exist for metastatic prostate cancer and conclusions are mainly drawn by analogy to breast cancer. Resel-Folkersma et al. showed that increased circulating tumor cells are associated with tumor progression and reduced survival and that removal of the prostate may therefore reduce the number of circulating tumor cells and prolong survival [45]. However, there are also indicators in breast cancer that removing the primary tumor might promote angiogenesis and increase metastatic ability and growth. As late as 1989, Fisher et al. demonstrated that following primary tumor removal, metastatic behavior may be affected by interplay of growth factors which can influence the outcome of a host to its tumor (Fisher effect) [46]. The observation that metastatic cancer recurrence may occur years to decades after therapy underlies the concept of tumor dormancy, a state of permanent minimal asymptomatic residual disease frequently found in breast and prostate cancer [47]. A number of physiologic processes as well as interventional procedures had been incriminated to cause the suspension of dormancy and thereby appearance of clinical metastases in several types of cancer [48]. Surgical removal of the primary tumor may stimulate distant metastases to proliferate and/or may elicit angiogenesis [49–51]. First, surgical manipulation of the tumour and its vascular supply may mechanically introduce circulating cancer cells into the circulation [52]. Second, removal of the primary tumor, which may be a source of antimetastatic and antiangiogenesis factors, may disturb the equilibrium and accelerate the metastatic and angiogenic process (Folkman effect) [53]. Controversies between the abscopal effect and the Fisher and Folkman effects in breast and other cancer types should be taken into account as a cautions warning in future metastatic prostate cancer trials. However, recent studies suggest that breast and prostate cancers may follow different paths. Badwe et al. have presented data from their prospective randomized controlled trial that definitive treatment of the primary breast tumor and axillary lymph nodes does not significantly affect survival in women with metastatic breast cancer who respond to neoadjuvant chemotherapy [54]. By analogy, local control in metastatic prostate cancer should not affect survival except in a small number of patients not responding to ADT. Furthermore, in a retrospective study published by Scodan et al., local control in metastatic breast cancer was associated with improved survival particularly marked in patients with widespread and visceral metastases [55]. Of note, the SEER database study of Culp et al. lacks information regarding the extent of bony metastasis and LND [35]. Including only oligometastatic patients in prospective randomised trial could also be a shortcoming and raises the importance of pertinent patient selection criteria before enrolment. However, a growing body of genetic and clinical literature suggests that treatment for metastatic prostate cancer may follow a different path compared to metastatic breast cancer. In a novel study, Haffner et al. tracked the clonal origin of lethal prostate cancer in a 64-year-old man who succumbed to metastatic disease 17 years after radical prostatectomy. Whole-genome sequencing was performed on three metastatic lesions procured at autopsy. Surprisingly, the lethal clone was tracked to a small focus of Gleason 3 disease in the primary tumor, not bulkier, higher-grade disease, or the early pelvic node metastasis [56].

### 3.3. Contemporary Role and Clinical Outcome of Local Treatment of Oligometastatic Prostate Cancer

#### 3.3.1. Oligometastatic Prostate Cancer: Is There a Definition?

Metastatic prostate cancer is clearly a heterogeneous entity with a wide spectrum of different malignant progression and aggressiveness ranging from the presence of circulating tumor cells in the blood to widespread polymetastatic disease. In 1995, it was suggested that the evolution of metastatic disease has intermediate states in which metastases might be present in limited numbers and sites, termed oligometastases [5]. The definition of oligometastatic state in prostate cancer is based, in current literature, on the extent of lymph node and bone involvement. However no clear definition exists. Singh et al. defined oligometastatic prostate cancer as five or fewer sites due to the more favorable outcomes seen in these patients and this definition was used in a subsequent number of trials [57]. However, recently, the metastatic site was defined as an important predictor of survival. Lymph node metastases alone, bone metastases alone or in association with lymph node, lung metastases, and liver metastases were associated with a median OS of 27.0 months, 20.3 months, 16.5 months, and 12.1 months, respectively [58]. These new data may help to establish a new definition and may contribute to treatment decisions in the design of future clinical trials for metastatic prostate cancer.

#### 3.3.2. Early Detection and Local Treatment of Oligometastatic Prostate Cancer: What Is the Rational and What Is the Benefit?

Recent developments of molecular and clinical imaging such as [11C] choline positron emission tomography (PET/CT), PET/MRI, 18 fluorodihydrotestosterone PET, (68) Ga-labelled prostate-specific membrane antigen (PSMA), combined ultrasmall superparamagnetic particles of iron oxide-enhanced, and diffusion-weighted (USPIO-enhanced MRI) and ferumoxytol enhanced MRI which had better specificity and sensitivity compared with anatomic imaging modalities such as CT and MRI are providing clinicians with the opportunity to detect lymphatic and/or haematogenous prostatic metastases at an earlier point in the disease progression, resulting in a treatment window for oligometastatic disease [59–63]. Consequently, the duration between a PSA rise and the detection of metastatic disease is decreasing as well as the number of metastases. A subset of patients classified as having a biochemical recurrence or with locally advanced disease would be classified as oligometastatic. This new state translates into an increased number of patients with oligometastatic disease in day to day practice. Controversies associated with the therapeutic management of this new state make the problem more apparent. Several options to eradicate oligometastases are being increasingly used. Schick et al. used, for example, high dose external beam radiotherapy combined with a short term ADT to treat isolated regional or distant metastases [64]. It is noteworthy to mention that there are no prospective trials on overall survival for the primary control of oligometastases. However, prospective randomized trials do exist and recent data are promising with encouraging biochemical-free survival, cancer control, and delayed time to ADT. The question whether the gain in time to progression with primary control of oligometastases represents an actual patient benefit or progression occurs at a fixed time point in the natural course of the disease, in which the delayed time to progression is explained only by early detection, remains to be answered. However, the hypothesis that eradication of small number of metastatic lesions might yield improved systemic control for oligometastatic cancer was generated several years ago. This is clearly the case for other cancers since curative surgical resection of liver metastases from colon cancer [65], lung metastases from a variety of primary sites [66], and adrenal metastases from lung cancer [67] result in improved overall survival and even cure in some patients. In prostate cancer, this concept might also be valid as the OS of patients with metastatic disease varies as a function of the number and sites of metastatic lesions as well as the number of metastases at recurrence [58]. Patients with initial low-volume metastatic disease were more likely to progress locally instead of distant, while the opposite was true for patients with high-volume metastatic disease [68]. That is why systemic treatments were proposed in clinical trials to treat polymetastatic cancer and aggressive local treatments were proposed for oligometastatic disease. This could be a shortcoming especially that, in analogy to metastatic breast cancer, patients with polymetastatic and visceral disease had the best response.

#### 3.3.3. Primary Control of Oligometastatic Prostate Cancer: What Is the Clinical Evidence?

While ADT is the current standard of treatment for patients diagnosed with symptomatic metastatic prostate cancer, its use in these patients with asymptomatic oligometastatic prostate cancer is controversial. The recent evidence of the potential toxic nature of ADT and its impact on quality of life has resulted in the advice that, as an alternative to immediate ADT, clinical surveillance can be suggested in patients with low-volume metastatic disease in order to defer ADT. However, surveillance is often associated with no treatment related to significant anxiety and uncertainty and besides deferring ADT related side effects, it had no positive impact on survival. Primary control of oligometastases had appeared as an interesting treatment modality to offer for these patients in clinical trials and two modalities are gaining interest in novel literature with encouraging results: salvage lymph node dissection (SLND) for patients with clinical lymph node relapse and salvage stereotactic body radiotherapy (SBRT) for patients with limited prostate cancer metastases.

*(1) Salvage Stereotactic Body Radiotherapy and In-Field Control of Oligometastases in Prostate Cancer*. The American Society of Radiation Oncology defines SBRT “as external beam radiotherapy used to deliver a high dose of radiation very precisely to an extracranial target within the body, as a single dose or a small number of fractions” [69]. This mini-invasive procedure increases the accuracy of treatment delivery thus reducing the amount of normal tissue irradiated. The high radiation dose per treatment SBRT can potentially ablate all tissues in the treated area [69]. Published studies of SBRT for oligometastatic prostate cancer can be divided into two types: first, studies in which a wide range of metastatic locations are treated independently of the primary tumor including prostate cancer [70]; second, those in which a single metastatic prostate cancer site is treated, such as the bone and/or lymph node (cf.  Table 1). The first studies addressing salvage SBRT for oligometastatic prostate cancer were published recently. Jereczek-Fossa et al. reported on 14 patients with isolated lymph node recurrence from prostate cancer treated with CyberKnife image-guided stereotactic radiotherapy [71]. Patients were staged and followed by choline PET/CT. At the mean follow-up of 18.6 months, the in-field control rate was 100% but five patients experienced clinical out-field progression. Later, the authors confirmed their CyberKnife based stereotactic radiotherapy approach in a larger homogeneous series [72]. In another study on patients with prostate cancer and nodal relapse alone on choline PET-CT scan, the 3-year treated metastases control rate was 90% after SBRT but DFS was only 17%. Lymph node recurrences were noted in 8 patients, all in sites outside the irradiated areas and 2 patients experienced early bone metastatic spread. Median PSA velocity was significantly lower in PET-negative patients compared to PET-positive subjects (0.40 ng/mL/year versus 2.88 ng/mL/year) [73]. Muacevic et al. reported on the use of SBRT for bony metastatic prostate cancer lesions many of which were spinal, but metastases in the skull, pelvis, and hip were also included. The study included 64 bony metastases in 40 patients, all treated with single-fraction SBRT. With a mean follow-up of 14 months, the authors report excellent response rates, with 6-, 12-, and 24-month estimates of local control of 95.5% [74]. The first study reporting on hormone naïve metastatic prostate cancer (bone and/or lymph node) was published by Berkovic et al. with a primary end point to defer systemic treatment; 10 patients out of 24 treated with a median follow-up of 24 months started with ADT resulting in a median ADT-FS, defined as the time interval between the first day of SBRT and the initiation of ADT, of 38 months [75]. The 2-year local control and clinical PFS were 100% and 42%, respectively. Moreover, repeated salvage SBRT in 14 patients for metachronous low-volume metastatic disease was feasible and well tolerated [75]. Similar findings on repeated salvage SBRT were also reported on a larger series published by Decaestecker et al. [76]. The authors identified PSA DT as the only variable influencing clinical progression and ADT-FS. The median PFS was 12 months for patients with a DT ≤ 3 months compared to 21 months for patients with a longer DT (*P* = 0.016). The median ADT-FS for patients with a PSA DT ≤ 3 mo was 18 months compared to 39 mo for patients with a longer DT (*P* = 0.014). In another study reporting on a cohort of patients with oligometastatic disease and detectable PSA, 100% achieved local control with SBRT to the metastatic lesions, and over half the patients achieved an undetectable or declining PSA by a median follow-up of 4.8 months even in mCRPC [77].

In view of these results, SBRT have demonstrated excellent local control with reported rates of 100% for nodal metastases and >90% for bone metastases. Both biochemical and clinical progression can be, at least temporarily, slowed down with a median time to clinical progression of 1–3 years in contemporary series. More interestingly, the pattern of recurrence appeared to be oligometastatic in 50% of the patients, allowing retreatment with SBRT. The tolerability of primary salvage and repeated salvage SBRT is excellent without significant grade 3 toxicity in the majority of the studies (cf.  Table 1). Oncological outcomes are also promising. Recently, Corbin et al. expanded on this concept suggesting the development of a specific oligometastatic phenotype over the natural course of a cancer's evolution that is less aggressive than other metastatic phenotypes [78]. This theory has been corroborated by microRNA analysis of clinically limited metastatic disease that accurately characterizes which patients will remain oligometastatic and which patients will proceed to polymetastatic disease [79]. Larger studies with more homogeneous patient populations are required to define the potential benefits of SBRT in the setting of prostate cancer. In addition, there are, currently, several ongoing trials on the treatment of oligometastatic prostate cancer with SBRT and/or cytokines. Limited data in the literature show a synergistic effect confirming more and more the efficacy of this treatment in advanced malignancies. Further research is needed to determine the potential impact of SBRT on systemic prostate cancer disease when combined with immunostimulating agents such as sipuleucel-T or Il 2 [80–82].

*(2) Salvage Lymph Node Dissection for Clinical Lymph Node Relapse after Local Curative Treatment of the Prostate*. The decision to start local or systemic therapy after BCR is a challenging process even for experienced physicians. A correct diagnosis of the site of prostate cancer recurrence is essential in the clinical decision making process in order to start targeted treatment instead of treating elevated PSA levels. However, in clinical practice, treatment is based on variables such as PSA DT, Gleason score, time from surgery to BCR, and surgical margins in the absence of an accurate imaging technique. These limitations explain the wide range of outcomes after BCR with some men progressing to overt metastatic disease and death despite therapy and others dying of other causes even without further prostate cancer intervention [83]. Recent developments in molecular and clinical imaging had led to the identification of a new group of patients with systemic disease progression, which is limited to the regional and/or retroperitoneal lymph nodes, termed clinical lymph node relapse. Furthermore, current imaging modalities contribute also to planning a personalised therapeutic strategy for these patients as demonstrated by the study of Colombié et al. [84]. They reported a significant change from palliative to curative treatment in 51.5% of their patients [84]. It is hypothesized that clinical lymph node relapse could be the direct consequence of a suboptimal PLND at the initial treatment or a progression outside the boundaries of the standard extended template. Recently, it was shown that these patients have a more favorable outcome compared to patients with progression to bone or to other organs [85]. In addition, it is well known that extended PLND or external beam radiation offers favorable cancer control outcomes especially in patients with microscopic limited lymph node invasion [86, 87]. A recently published randomized trial showed that, at a median follow-up of 74 months, extended PLND (in comparison with standard PLND) can significantly improve the BCR-free rate by 13% and 20% in patients with intermediate- and high-risk prostate cancer, respectively [58]. For node positive disease without signs of distant metastases at the time of local therapy, there is currently no consensus regarding the optimal timing for ADT. The quality of data is low and available evidence suggests a small improvement in survival and delayed disease progression but increased adverse events in the group treated with early ADT compared to the deferred ADT group [88]. In contrast, a large observational population based study found no survival benefit deferring immediate ADT in men with positive lymph nodes after radical prostatectomy [89]. Furthermore, Dale et al. demonstrated in a prospective cohort of old men that patient anxiety independently predicts early initiation of ADT for BCR [90]. In order to avoid ADT, some authors use 5*α*-reductase inhibitors to reduce PSA and anxiety [91]. Salvage LND had also been used in these patients [92–94]. A recent systematic review highlights the promising results of such an approach [95]. Immediate complete biological response, defined as a PSA < 0.2 ng/mL, was found in 40.7%–59.3% of patients. The response was durable in subsequent 9–29.4% of these patients at 5 years of follow-up. In series with follow-up >5 years, one-third of these patients remained free of clinical recurrence. The 8-year CSS rate was 80.6% in one large study and all other studies reported excellent 5-year CSS rates (75%–89.1%) (cf.  Table 2). Winter et al. analysed a select group of patients with a single positive spot at PET/CT scan treated with SLND [96]. All metastasis-suspicious LNs at PET/CT scan were histologically confirmed and all other removed LNs were negative for disease. The authors concluded that only suspicious nodes on choline PET/CT scan should be removed. However, these findings were not confirmed in a subsequent report that examined a substantially larger sample size and observed that LN metastasis frequently involves nodes other than those detected by PET/CT scan [97, 98]. Furthermore, the nodes detected by PET/CT scan might be negative in up to 25% of cases [99]. The common association of adjuvant treatment combined with SLND in patients with a PSA response limits the interpretation of midterm cancer control outcomes. In one of the largest series reported to date, 32% of patients received adjuvant hormonal therapy [100]. Of patients with complete biochemical response and no adjuvant hormonal therapy, a further PSA progression was observed in 86% of patients [100]. This finding corresponded to a 5-year BCR-free survival rate of 19% compared to 34% in the entire cohort [100]. In the latter study, a complete PSA response following SLND was noted in 30% of patients followed up for 5 years. However, the percentage of men who were treated with adjuvant hormonal therapy was not indicated in this cohort [100]. Finally, in the only multi-institutional report available to date, Suardi et al. examined the data from five tertiary referral centres of 162 patients affected by BCR after radical prostatectomy associated with nodal recurrence detected at either 11C-choline PET/CT scan or conventional imaging. A total of 132 patients (81%) were found to harbor a pathologically confirmed clinical LN relapse, and 66 patients (41%) achieved a complete biochemical response after surgery [101]. Several postoperative factors, including complete biochemical response, Gleason score, the location of positive LNs at SLND, and the number of positive LNs at SLND, were established as independent predictors of clinical progression in these studies [94]. In view of these data, SLND might represent a therapeutic option for very well-selected patients. Safety and efficacy should be tested in further randomized control trials. Patients with PSA value <4 ng/mL, Gleason score <8, and a clinical LN relapse limited to the pelvis only and well informed motivated patients might represent the ideal candidates for inclusion in these trials.

## 4. Conclusion

Since the definition of an oligometastatic state of cancer, a paradigm shift toward more aggressive local control of primary tumor and/or small number of metastatic lesions and/or clinical LN relapse is gaining interest in many oncologic centres. Local control of primary tumor in metastatic prostate cancer remains experimental with only one retrospective observational study showing prolonged survival. However, a growing body of genetic and molecular research supports these findings. Several options to eradicate oligometastases exist with excellent local control rates. SBRT has been demonstrated as a safe, well tolerated, and efficacious treatment for lymph node and bone lesions. Both biochemical and clinical progression are slowed down with a median time to initiate ADT of 2 years. SLND is feasible in patients with clinical LN relapse after local curable treatment and can be used in future prospective randomized trials. Despite encouraging midterm results, a complete cure remains elusive in these patients denoting the importance of an optimal initial PLND. Further advances in imaging are crucial in order to rapidly evolve beyond the proof of concept.

## Figures and Tables

**Table 1:** Stereotactic body radiotherapy for oligometastatic prostate cancer.

Author	Year	Number of patients (number of lesions)	Dose	Primary site	Treated site (s)	Local control	Toxicity	Remarks
Greco et al. [70]	2011	103 (126)	18–24 Gy in 1 fraction (SBRT)	Prostate, renal, and colorectal	Bone, LN, and soft tissue	64% (82% if >22 Gy, 25% for 18–20 Gy) at 2 years	<4% grade 3 (stricture, neuritis)	

Jereczek-Fossa et al. [71]	2009	14 (14)	30 Gy in 3 fractions (Linac-CyberKnife)	Prostate	Pelvic LN	100% at 18.6 months	No grade 3 or higher	

Casamassima et al. [73]	2011	25 (25)	30 Gy in 3 fractions	Prostate	Prostate, Pelvic LN, para-aortic LN, and mediastinal LN	90% at 3 years	No grade 2 or higher	DFS 17% at 3 years

Jereczek-Fossa et al. [72]	2012	34 (38)	30 Gy in 5 fractions to 36 Gy in 3 fractions (CyberKnife)	Prostate	LN and bone	88% at 16.9 months	6% grade 3 urinary and 3% grade 3 rectal	All toxicities seen in prostate recurrence patients

Muacevic et al. [74]	2013	40 (64)	20 Gy in 1 fraction (SBRT)	Prostate	Bone	95.5% at 2 years	No grade 3 or higher	

Berkovic et al. [75]	2013	24 (29)	50 Gy in 10 fractions (repeated SBRT)	Prostate	Bone or LN	100% at 2 years	No grade 3 or higher	DFS of 42% at 2 years

Ahmed et al. [77]	2013	17 (21)	20 Gy in 1 fraction (SBRT)	Prostate	Bone, LN, and liver	100% at 4.8 months	No grade 3 or higher	

Schick et al. [64]	2013	50 (50)	64 Gy (EBRT)	Prostate	Bone, LN, and visceral	—	No grade 3 or higher	BRFS, CFFS, OS of 54.5%, 58.6%, and 92%, respectively

Decaestecker et al. [76]	2014	50 (70)	50 Gy in 10 fractions or 30 Gy in 3 fractions (repeated SBRT)	Prostate	Bone and LN	100% at 2 years	Grade 1 (14%) Grade 2 (6%)	ADT-FS median 25 months

LN: lymph node; SBRT: stereotactic body radiotherapy; BRFS: biochemical recurrence free survival; CFFS: clinical failure free survival; OS: overall survival; ADT-FS: androgen deprivation therapy free survival; DFS: disease free survival.

**Table 2:** Salvage lymph node dissection for biochemical recurrence following radical prostatectomy.

Author	Year	Number of patients	Mean number of positive LNs (mean number of LNs removed)	Median follow-up, mo	Complete biologic response, %	Mean 5-yr BCR-free survival, %	5-year progression-free survival, %	5-year cancer-specific survival, %
Schilling et al. [94]	2008	10	2.8 (7.1)	—	—	—	—	—
Rinnab et al. [93]	2008	15	— (13.9)	13.7	—	—	—	—
Winter et al. [96]	2010	6	1 (10)	24	50	—	—	—
Rigatti et al. [99]	2011	72	9.8 (30.6)	39.4	56.9	19	34	75
Jilg et al. [92]	2012	52	9.7 (23.3)	35.5	46	9	26	78
Suardi et al. [101]	2013	162	6.1 (24.6)	29.2	40.7	40	47	86
Suardi et al. [100]	2014	59	8.9 (29.5)	81.1	59.3	29.4	52.0	89.1
Tilki et al. [98]	2013	56	5.1 (21)	—	—	—	—	—
